# Collision tumour of large-cell neuroendocrine carcinoma and adenocarcinoma in the stomach: A case report

**DOI:** 10.3332/ecancer.2016.616

**Published:** 2016-01-29

**Authors:** Eduardo Payet, Pau I Pilco, Jaime Montes, Alejandra Cordero-Morales, Maria Jose Savitzky, Karoline Stenning-Persivale

**Affiliations:** 1 Clínica Anglo Americana, Calle Alfredo Salazar S/N, San Isidro, Lima 18, Peru; 2 Clínica Delgado, Avenida Angamos Oesta cuadra 4, Miraflores, Lima 27, Peru; 3 Universidad Peruana de Ciencias Aplicadas, Prolongación Primavera 2390, Santiago de Surco, Lima 33, Peru

**Keywords:** collision tumour, carcinoma, neuroendocrine, adenocarcinoma

## Abstract

Concurrence of adenocarcinoma and large-cell neuroendocrine carcinoma of the stomach is a rare condition. Here, we report a case of gastric collision tumour with large-cell neuroendocrine carcinoma and adenocarcinoma. A 71-year-old Peruvian man presented with nausea, epigastric pain, and weight loss for seven months. An Endoscopic evaluation revealed a huge ulcerative and infiltrative mass in the upper and middle third of the stomach. The patient underwent a D2 total gastrectomy. Microscopically, two separated and attached ulcerative lesions were recognised. The proximal to the cardial lesion showed neuroendocrine morphology and immunoreactivity for synaptophysin, and the other a moderated tubular adenocarcinoma Borrmann type III. Both lesions invaded serosa and lymph nodes metastases were found in 17 of 41 lymph nodes retrieved (one lymph node with neuroendocrine metastatic deposits).

## Introduction

Adenocarcinomas are largely the most common gastric malignant tumour and concurrent or synchronous presentation in the same stomach of different histological type tumours rarely occurs. Lewin proposed a nomenclature for these neoplasms: (a) Composite tumours for the mixture of exocrine and endocrine components; (b) Collision tumours, where the exocrine and endocrine components differ and are juxtaposed and finally; (c) Amphicrine cell tumours, where the endocrine and exocrine cells constituents are present within the same cell, exhibiting dual differentiation [1, 2].

Collision tumours are neoplasms consisting of two distinct cell populations, developing in juxtaposition to one another, without areas of intermingling [3]. These types of tumours are believed to arise independently from a multipotential epithelial stem cell or a primitive neuroendocrine carcinoma, respectively, and it is coincidental that they exist next to each other [2, 4]. Here, we present a rare case of a gastric collision tumour displaying features of both large-cell neuroendocrine carcinoma (LCNEC) and adenocarcinoma.

## Case report

A 71-year-old man was admitted to our outpatient clinic, with nausea, epigastric pain, and weight loss that had lasted for seven months. Laboratory findings showed anaemia. The patient underwent an endoscopic evaluation, which revealed a huge ulcerative and infiltrative mass in the upper and middle third of the stomach and the biopsy specimen taken showed a moderated tubular adenocarcinoma. Contrast-enhanced computerised tomography (CT) scan revealed a thick engrossment of the two upper thirds of the stomach with enlarged perigastric lymph nodes and not liver, nor peritoneal metastases findings were shown. The patient underwent a D2 total gastrectomy with distal pancreatectomy and splenectomy. At surgery, the tumour was located in the upper two-thirds of the posterior wall of the stomach with enlarged lymph nodes at the lesser curvature and celiac trunk. There was serosal invasion and fixation to the anterior surface of the pancreas body. No liver or peritoneal metastases were found.

**Figure 1.**
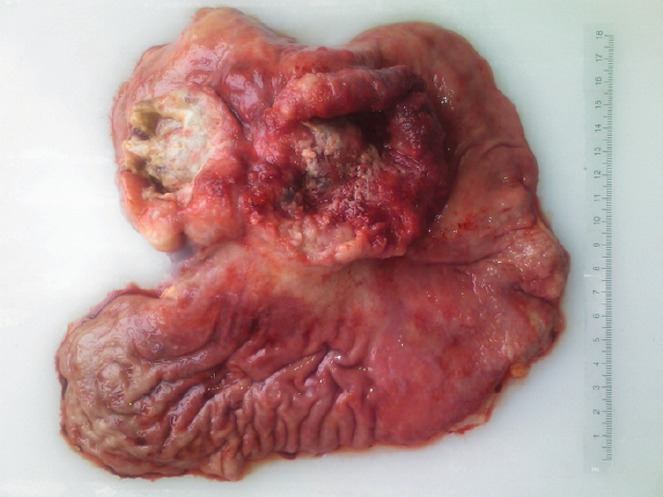
Resected specimen showing two attached ulcerative lesions in the posterior surface of the proximal and middle third of the stomach.

**Figure 2.**
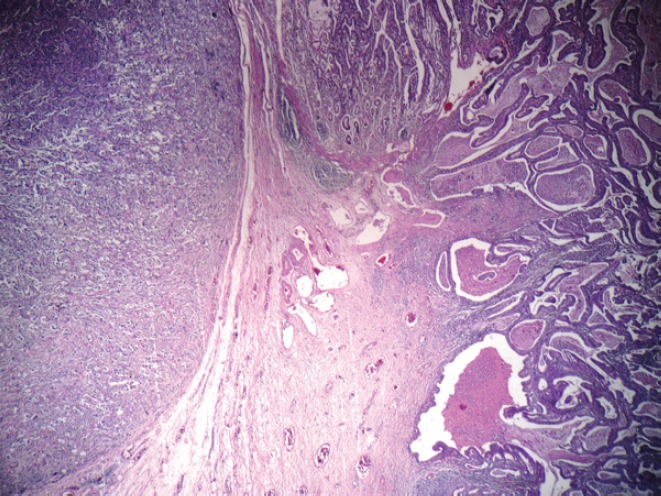
Two separated lesions showing both neuroendocrine carcinoma and tubular adenocarcinoma at H & E.

**Figure 3.**
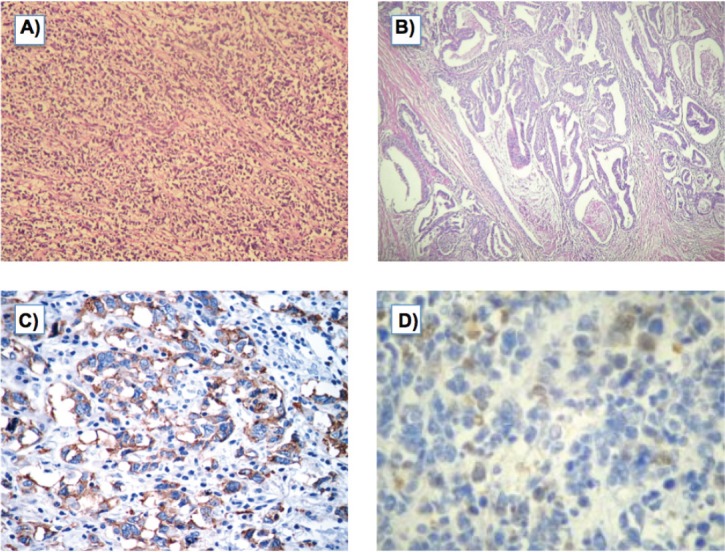
A) Neuroendocrine carcinoma (H & E), B) Moderated tubular adenocarcinoma (H & E), C) Tumour cells with neuroendocrine histology showing cytoplasmic inmunoreactivity for synaptophysin, D) Tumour cells with neuroendocrine morphology showing focal cytoplasmic inmunoreactivity to pankeratin.

On gross examination of the specimen, we identified two attached ulcerative tumours located on the posterior wall of the stomach (Figure 1). The proximal one close to the cardial region measured 6.5 × 5.5 cm, with a whitish grey coloration and with an elastic consistency; and closely attached, a fungating ulcerative reddish lesion (Borrmann type III) measured 8.5 × 8.0 cm and occupied the posterior surface of the middle third of the stomach. The latter fixedly attached to the anterior surface of the body of the pancreas. The spleen did not show alterations. In addition to the gastric specimen, 41 resected regional lymph nodes were submitted and studied. Microscopically, two separated gastric lesions were identified with different patterns in morphology and the distance in between was of 1 mm (Figure 2). The proximal lesion corresponds to a large-cell neuroendocrine carcinoma composed of atypical cells with faint cytoplasmic features and prominent hyperchromatic nuclei (occasionally pleomorphic). The cells are distributed in an organoid architecture with solid nests and broad trabeculae solid pattern without glandular formation (Figure 3A). The distal tumour corresponds to a moderated tubular adenocarcinoma, composed mainly of tubules infiltrating the gastric wall with serosal exposure. The cells contain variable amounts of eosinophilic cytoplasm with scattered mucin material (Figure 3B).

The neuroendocrine phenotype of the large-cell neuroendocrine carcinoma was immunohistochemically confirmed with synaptophysin, which stain all (Figure 3C). Additionally, it showed focal expression for keratin (AE1/AE3) (Figure 3D). Other markers, such us CD45, CD3, CD20, CD30, and S100, were negative.

The tumour cells invaded veins as well as lymphatic nodes. Regional lymph node metastasis was detected in 17 out of 41 lymph nodes. Sections showed adenocarcinoma in all lymph nodes retrieved, but only one lymph node showed metastasis from the neuroendocrine carcinoma.

## Discussion

Gastric large-cell neuroendocrine carcinomas (LCNECs) are a rare type of tumours that account for less than 1.5% of all types of gastric cancers and have a very poor prognosis [5, 6]. These are tumours identified by immunohistochemistry and electron microscopy due to its distinguishing features that indicates endocrine differentiation. Due to the low frequency of presentation, these tumours have not been well described [7].

On the other hand, adenocarcinomas account to up to 95% of all malignancies found in the stomach and are by far the most studied type of gastric tumour [8]. It is known that adenocarcinomas can frequently have a mixed composition with minor endocrine components inside the tumour; that is why the identification of a LNEC in addition to the original adenocarcinoma demonstrates the presence of two different tumours coexisting, instead of the initial thought of a poorly differentiated adenocarcinoma [9].

The prognosis of the patient will be determined by the aggressiveness and extension of the tumour with deeper invasion of the gastric wall and the proportion of lymph node metastases. In this case, the most aggressive lesion was the adenocarcinoma, which showed serosal exposure and great proportion of lymph node metastases. This tumour was classified as a stage IIIC, using the criteria established by the American Joint Commission on Cancer (TNM), indicating that surgery was the first choice of treatment [9]. The case was discussed with the patient, offering him the possibility of postoperative radiochemotherapy. However, the patient refused this option and he was given only palliative care.

## Conclusion

To our knowledge, there are fewer than 40 cases of reported collision tumours with the characteristics given [11, 12]. These types of tumours are rare entities diagnosed after surgery, with the use of pathology studies. Collision tumours are described as mixed tumours, which presents with two different cell populations that appear to arise as separate lesions juxtaposed to one another [10].

## Ethical statements

All procedures followed were in accordance with the ethical standards of the responsible committee on human experimentation (institutional and national) and with the Helsinki Declaration of 1964 and later versions. Informed consent or substitute for it was obtained from all patients for being included in the study.

## Conflicts of interest

The authors declare that they have no conflict of interest.
